# Antiviral activity of molnupiravir against COVID-19: a schematic review of evidences

**DOI:** 10.1186/s42269-022-00753-9

**Published:** 2022-03-10

**Authors:** Shivali Singla, Sachin Goyal

**Affiliations:** Department of Pharmaceutics, School of Pharmacy, Abhilashi University, Chail Chowk, HP 175028 India

**Keywords:** Molnupiravir, EIDD-2801, EIDD-1931, COVID-19, SARS-Cov-2

## Abstract

**Background:**

The study was aimed at encapsulating the evidence of in vitro and in vivo antiviral activities of molnupiravir and its active form against highly pathogenic SARS-CoV-2, the pathogen responsible for COVID-19, and finding out the efficacy and safety of molnupiravir in clinical trials.

**Main body:**

Information on publications was explored on several databases, gray literature was reviewed, and the outcomes were discussed narratively. Molnupiravir's antiviral efficacy and associated mechanism of action have been verified in vitro against both non-COVID and multiple coronaviruses. Molnupiravir has been tried in preclinical investigations in numerous animal models against non-coronaviruses. Clinical studies in several countries are now being conducted to evaluate its antiviral efficacy in persons infected with COVID-19. The medication displays antiviral effect via generation of copying mistakes during viral RNA replication.

**Conclusions:**

Molnupiravir is the first oral antiviral medicine to show considerable and convincing antiviral activity in vitro and in animal models. Molnupiravir stops the spread of SARS-CoV-2 in animals that have been infected and in cells grown in a lab. In a clinical research, early molnupiravir treatment reduced hospitalization and death risk in unvaccinated individuals with COVID-19. In the battle against SARS-CoV-2, it could be a potent weapon. However, its role in COVID-19 in moderate to severe cases is still up in the air, and more research is needed.

## Background

During the global pandemic of Coronavirus Disease 19 (COVID-19), which was brought on by the severe acute respiratory syndrome coronavirus 2 (SARS-CoV-2), there was a highly transmittable and pathogenic viral infection, that led to a theatrical loss of human life worldwide. An outbreak of the novel coronavirus affected Wuhan city, China, at the end of 2019. Within fifty days of the epidemic, over eighteen hundred individuals died and over seventy thousand were infected (Shereen et al. 2020). The virus is believed to be a member of the β group of coronaviruses. The Chinese experts named the new virus 2019 novel coronavirus (2019-nCov) by the Chinese experts. This virus was identified as SARS-CoV-2 by the International Committee on Taxonomy of Viruses (ICTV) and the disease as COVID-19 by WHO (World Health Organization) (Cui et al. 2019; Knight 2020; Organization 2020a; Qi 2020). Since then, the disease has rapidly circled the globe and has eventually affected every continent including Antarctica. It has been categorized as a pandemic by the World Health Organization (Organization 2020b; Chakraborty and Maity 2020). Due to the most crucial global health disaster of the current century, national healthcare systems are faced with significant challenges in affected countries (Rodriguez-Morales et al. 2020).

However, people of all ages get infected by COVID-19, though attention needs to be given to those at a greater risk of developing severe illness, such as the elderly and people with underlying comorbidities e.g. diabetes mellitus, cardiac disorder, respiratory disease, chronic liver diseases, and renal impairment. Patients with cancer and those utilizing immunosuppressants, as well as pregnant women, are also believed to be at a higher risk of developing severe disease if infected (Azer 2020; Wang et al. 2020). The most common symptoms are respiratory stress, including fever, dry cough, dyspnea, shortness of breath, and Computed Tomography imaging (CT) demonstrated ground-glass opacities as the most frequent outcome (Ali and Alharbi 2020; Sahu et al. 2020; Parekh et al. 2020). In addition to these, COVID-19 could also affect the gastrointestinal system along with the liver (Mao et al. 2020), cardiovascular system (Mishra et al. 2020), kidneys (McAdams et al. 2021; Amann et al. 2021), nervous system (Iadecola et al. 2020) and other organs (Benny and Khadilkar 2020; Harapan et al. 2020).

Anti-SARS-CoV-2 treatment for patients with a suspected or confirmed COVID-19 infection hasn't been shown to be effective by randomized controlled trials so far (Jean et al. 2020; Zhai et al. 2020; Cascella et al. 2021; Emani et al. 2021). This means that there's a big need for new treatments that target SARS-CoV-2. However, several treatment strategies have been adapted depending upon the severity of the infection, including immune-boosters from therapies such as Unani or Ayurveda (Khanna et al. 2021). The National Health Commission and State Administration of Traditional Chinese Medicine guidelines, diagnosis, and treatment protocols for novel coronavirus pneumonia (7th edition) recommend several treatments for COVID-19, including RNA dependent RNA polymerase inhibitors (lopinavir/ritonavir combination), arbidol, favipiravir, remdesivir, and other antivirals such as interferon α (IFN-α) (Tobaiqy et al. 2020; Ghanbari et al. 2020; Tian et al. 2021). There is strong evidence that two antimalarial drugs, chloroquines and hydroxychloroquine, can fight SARS-CoV-2 in the lab (Prevention 2020; Khuroo 2020). Immunosuppressive corticosteroids (Dexamethasone or Prednisolone), monoclonal antibodies, and convalescent plasma therapies are also effective (Stasi et al. 2020).

Several mechanisms are used by these small molecule broad-spectrum antivirals to exert their effect, including blocking viral entry, activation of inactive enzyme(s), blocking the formation of virus particles, or targeting a host factor involved in replication (Zumla et al. 2016). However, no selective coronavirus antivirals have been approved to date to completely cure or prevent coronavirus infections, and the approved ones, such as remdesivir, only weaken viral loads to the extent that they reduce the chance of disease severity (Abdelnabi et al. 2021), with the limitation of administration via IV route (Humeniuk et al. 2020; Martinot et al. 2020).

### Molnupiravir: an introduction

**Fig. 1:**
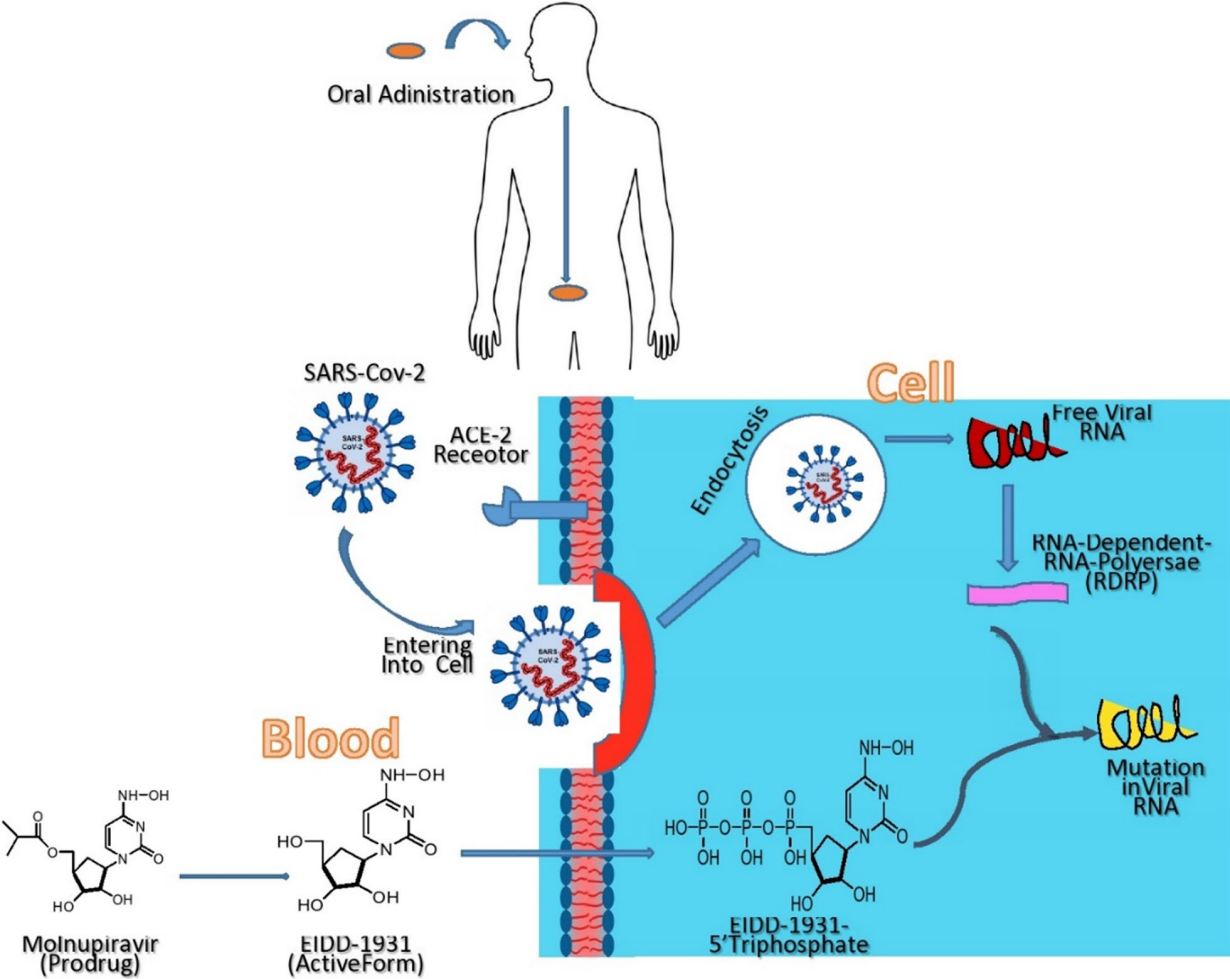
Mechanism of molnupiravir and its pharmacological active form after oral administration

**Fig. 2:**
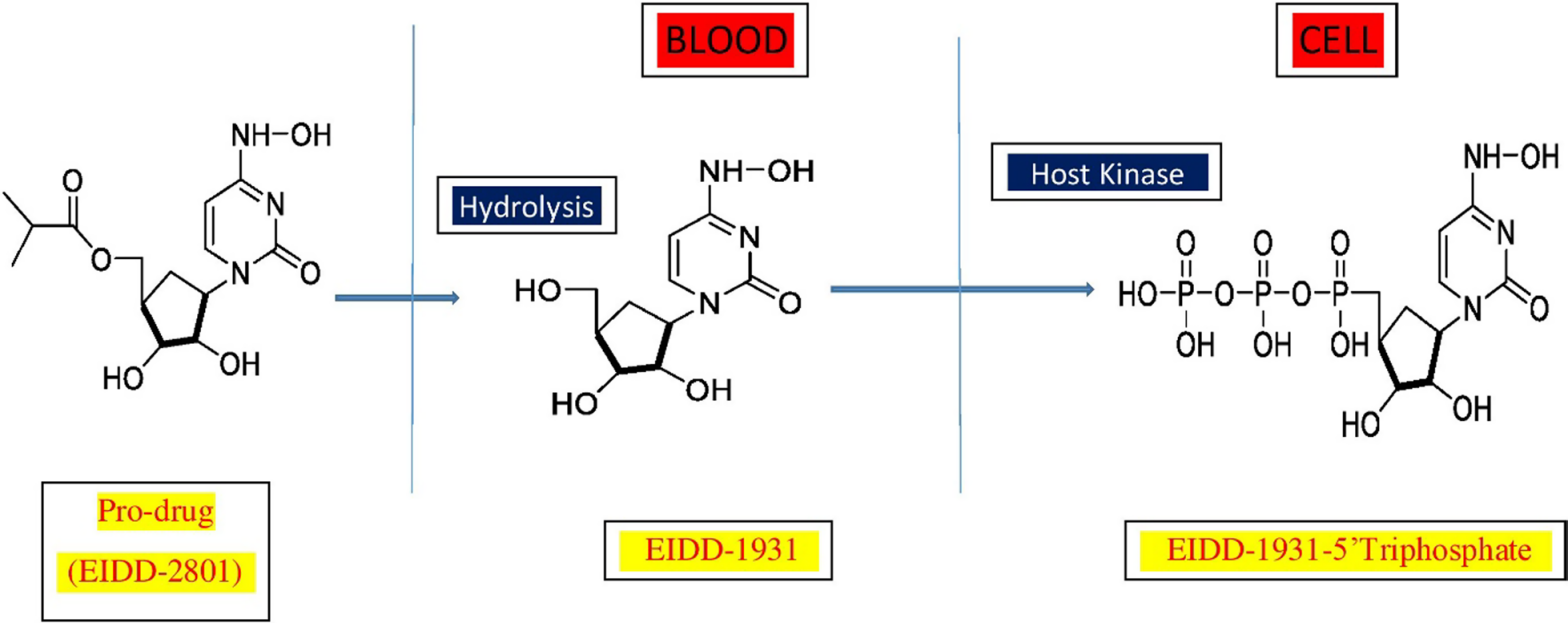
Molnupiravir converted into EIDD-1931 in blood then in cell by host kinase converted into EIDD-1931-5’Triphosphate (active form)

Molnupiravir (MK-4482 and EIDD-2801) is an orally active investigational antiviral medication that was developed to treat hepatitis and influenza (Toots et al. 2019, 2020). It's an isopropylester prodrug (molecular formula C_13_H_19_N_3_O_7_, molecular mass 329.31 g·mol^−1^) of the synthetic nucleoside derivative N4-hydroxycytidine, which gets hydrolyzed in vivo to an intermediate EIDD-1931 (NHC or -DN4-hydoxycytidine) and is allocated to tissues, where it's transformed into an active 5'-triphosphate by host kinase (Figs. 1 and 2). It works against viruses by introducing copying errors during viral RNA replication, a process known as viral error catastrophe. (Agostini et al. 2019; Painter et al. 2019; Huchting 2020; Gordon et al. 2021). The drug was developed at Emory University by the university's drug innovation company, Drug Innovation Ventures at Emory (DRIVE), which was acquired by a Miami-based company, Ridgeback Bio Therapeutics, which later merged with Merck & Co (Wikipedia contributors). This orally bioavailable, potent ribonucleoside analog and its active form (NHC) were previously reported to inhibit diarrhoea virus & hepatitis C virus (Stuyver et al. 2003), norovirus (Costantini et al. 2012), chickungunya virus (Ehteshami et al. 2017), Ebola virus (Reynard et al. 2015; Schafer et al. 2018), influenza viruses and syncytial viruses (Yoon et al. 2018; Toots et al. 2019; Toots and Plemper 2020), CoV (Agostini et al. 2019), and Venezuelan equine encephalitis virus (VEEV) (Urakova et al. 2018), coronaviruses including SARS, MERS, and SARS-CoV-2. Remdesivir-resistant mutant mouse hepatitis virus has also been shown to have increased sensitivity to N^4^-hydroxycytidine (Sheahan et al. 2020; Şimşek Yavuz and Ünal 2020). Earlier the drug was reported for its mutagenic effect on multiple bacterial systems (Salganik et al. 1973; Popowska and Janion 1974, 1975). In order to treat severe acute respiratory syndrome coronavirus 2 (SARS-CoV-2) and other kinds of coronaviruses, it has been repurposed and is currently in phase II/III clinical trials.

The present review is aimed at assessing the potential of molnupiravir in the prevention and treatment of patients diagnosed with SARS-CoV-2 by incorporating results from laboratory studies, in vivo experiments, and clinical trials.

## Main text

### Material and methods

Appropriate publications were explored on Science Direct, PubMed, Nature, Springer, and Google Scholar using the terms "Molnupiravir", or "EIDD-2801", or "EIDD-1931", or "NHC" and in the heading and abstract or main text, including "COVID-19", "SARS-CoV", "MERS-CoV", and "SARS-CoV-2". The Clinical Trials.gov databank was searched to find ongoing and completed clinical trials evaluating the efficacy of molnupiravir against COVID- 19. The screened articles were categorized and well-ordered and examined based on the study types: Mechanism of Molnupiravir, In vitro, and In vivo.

## Results

A total of 21 leading references are associated with molnupiravir from all articles reviewed between May 20, 2021 and the present date. In vitro studies have confirmed the antiviral activity and related mechanism of molnupiravir in both non-COVID and multiple coronaviruses (Stuyver et al. 2003; Hollecker et al. 2004; Costantini et al. 2012; Reynard et al. 2015; Ehteshami et al. 2017; Urakova et al. 2018; Yoon et al. 2018; Agostini et al. 2019; Toots et al. 2019, 2020; Sheahan et al. 2020; Toots and Plemper 2020; Rosenke et al. 2021). Molnupiravir has been tested in preclinical studies on non-coronaviruses in several animal models (Yoon et al. 2018; Agostini et al. 2019; Toots et al. 2019; Sheahan et al. 2020; Sticher et al. 2020; Abdelnabi et al. 2021; Wahl et al. 2021). Clinical trials are ongoing to assess its antiviral effects in humans with COVID-19 in different countries (Emani et al. 2021).

## Discussion

### In vitro* investigation*

The active form, β-DN^4^-hydoxycytidine (NHC), exhibited antiviral activity in both the BVDV and the HCV replicon cell cultures (Stuyver et al. 2003; Hollecker et al. 2004). Anti-noroviral activity of NHC was reported in norovirus replicon cell cultures, showing an EC50 value of 1.5 µM (Costantini et al. 2012). Olivier Reynard el al investigated the activity of NHC to obstruct viral replication as well as its potential cytotoxicity over an extensive concentration range in both VeroE6 cells infected with Ebola virus and primary macrophages that designate a critical cellular goal for Ebola virus replication. The values of EC50 were 3 and 3.8 µM for inhibition of transcription and virus spread, respectively (Reynard et al. 2015). In a study in 2017, NHC was identified, characterized, and reported to influence chickungunya virus in four types of cultures: Huh-7–CHIKV, BHK-21–CHIKV, and two types of CHIKV infectious model strains with EC50 values between 1.8 to 0.2 µM (Ehteshami et al. 2017). In this study, NHC was described as a potent anti-VEEV agent, with an EC50 below 1 μM and found more effective in the first 4 h post infection (Urakova et al. 2018). For influenza A and B viruses in cultures of well-differentiated human air–liquid interface airway epithelia relevant to disease, inhibitory concentrations for NHC were found to be very low, with EC50 values of 0.06 to 0.08 μM (Toots et al. 2019). During the COVID pandemic, in vitro studies showed that the drug inhibited both coronavirus murine hepatitis virus (MHV) and Middle East respiratory syndrome CoV (MERS-CoV) with minimal cytotoxicity (EC 50 summarized in Table 1). It inhibited MHV lacking ExoN proofreading activity similarly to wild-type (WT) MHV, suggesting an ability to evade or overcome ExoN activity, pointing to a virus-mutagenic mechanism of NHC inhibition in CoVs and indicating a high genetic barrier to NHC resistance (Agostini et al. 2019). The antiviral activity of NHC against MERS-CoV in Calu-3 2B4 ("Calu-3" cells) was remarkable in recent studies (Rosenke et al. 2021) done on cell lines infected with MERS-CoV and the recently emerged SARS-CoV-2 (Table 1) with no perceived cytotoxicity. A clinical isolate of SARS-CoV-2 (2019-nCOV/USA-WA1/2020) was tested in African green monkey kidney (Vero) cells, and its antiviral activity was demonstrated in Calu-3 cells as well (Sheahan et al. 2020; Rosenke et al. 2021). Primary human airway epithelial (HAE) cell cultures were next assayed using the drug, which revealed anti-SARS-CoV and SARS-CoV-2 activity. These findings establish NHC as an effective antiviral against SARS-CoV-2, MERS-CoV, SARS-CoV, and zoonotic bat-CoVs, and call for a strong stand against COVID-19 (Sheahan et al. 2020).

**Table 1:** In vitro studies on efficacy of NHC (Active component of Molnupiravir) against highly pathogenic Coronaviruses (MHV-A59, MERS-CoV, SARS-CoV, and SARS-CoV-2)

S. no.	Drug molecule	Cell line	Virus	IC50 & EC 50	References
1.	EIDD-1931	Murine astrocytoma delayed brain tumor cells	Coronavirus MHV-A59	EC50 = 0.17 μM	Agostini et al. (2019)
2.	EIDD-1931	Vero cells	MERS-CoV	EC50 = 0.56 μM	Agostini et al. (2019)
3.	EIDD-1931	Calu-3 2B4	MERS-CoV	IC50 of 0.15 μM	Sheahan et al. (2020)
4.	EIDD-1931	Vero E6 Cells	SARS-CoV-2	IC50 of 0.3 μM	Sheahan et al. (2020)
5.	EIDD-1931	Calu-3	SARS-CoV-2	IC50 of 0.09 μM	Sheahan et al. (2020)
6.	EIDD-1931	HAE	SARS-CoV-2		Sheahan et al. (2020)
7.	EIDD-1931	HAE	MERS-CoV	IC50 = 0.024 μM	Sheahan et al. (2020)
8.	EIDD-1931	HAE	SARS-CoV	IC50 = 0.14 μM	Sheahan et al. (2020)
9.	EIDD-2801	Calu-3	SARS-CoV-2	IC50 = 414.6 nM	Rosenke et al. (2021)

Coronavirus MHV-A59: coronavirus murine hepatitis virus A-59; Vero E6: African green monkey kidney epithelial cells; MERS-CoV: Middle East respiratory syndrome-related coronavirus; Calu-3: human bronchial epithelial cells; SARS-CoV-2: The virus, severe acute respiratory syndrome coronavirus 2; HAE: human airway epithelial cells; SARS-CoV: severe acute respiratory syndrome-related coronavirus

Other antiviral like remdesivir and biological agents (convalescent plasma or monoclonal antibodies), which require infusion during a clinical visit, suggest the benefits of molnupiravir for a broader distribution, being an orally efficacious agent and being more acceptable to the patient (Kim et al. 2020). Molnupiravir has also been shown to be effective against additional versions of SARS-CoV-2, including as the Omicron form, both alone and in combination with nirmatrelvir (Li et al. 2022). Other variations such as alpha, beta, gamma, and delta were also shown to be molnupiravir-susceptible (Vangeel et al. 2022).

### In vivo investigations

EIDD-2801 has displayed promising anti-influenza and anti-syncytial virus activity in various animal models, viz. mice, guinea pigs, cynomolgus macaques, and ferrets (Yoon et al. 2018; Toots et al. 2019, 2020). A pharmacological treatment with Molnupiravir (EIDD-2801) twice daily of infected animals (ferret) significantly decreased the SARS-CoV-2 load in the upper respiratory track and effectively prevented spread to cohoused animals. Sheshan et al. revealed that prophylactic and therapeutic consumption of EIDD-2801 in mouse models significantly reduces MERS-CoV replication, thus reducing lung viral loads and pathogenesis coincident while increasing mutation rates of viral proteins (Sheahan et al. 2020). According to other research, oral EIDD-2801 may disrupt SARS-CoV-2 public transmission chains (Cox et al. 2021). In Syrian hamsters given EIDD-2801 for four consecutive days starting on the day of infection, a dose-dependent reduction in lung viral loads was observed along with improved lung histopathology scores (Abdelnabi et al. 2021). In grafted human lung tissue in the LoM (lung-only mice that are immune-deficient) model, malnupuiravir, an orally effective drug, was found to strongly inhibit SARS-CoV, SARS-CoV-2, MERS-CoV, and SARS-like bat coronavirus replication after therapeutically administered, and while administered as pre-exposure prophylaxis, it also barred SARS-CoV-2 infection, strongly supporting the fact of further clinical development of antiviral drug for COVID-19 (Wahl et al. 2021). In recent studies, Molnupiravir (MK-4482) was exposed to inhibit SARS-CoV-2 replication in the Syrian hamster model, which demonstrated the ability to control SARS-CoV-2 spread and also for the treatment of COVID-19 (Rosenke et al. 2021) (Table 2).

**Table 2:** In vitro studies on efficacy of molnupiravir against highly pathogenic Coronaviruses

S. no.	Drug molecule	Animal or animal model	Virus	References
1.	EIDD-2801	Ferret Model	SARS-CoV-2	Yoon et al. (2018)
2.	EIDD-2801	Mouse model	MERS-CoV	Sheahan et al. (2020)
3.	EIDD-2801	LoM Model	SARS-CoV-2 MERS-CoV SARS-CoV SARS-like bat coronaviruses	Wahl et al. (2021)
4.	EIDD-2801	Syrian Hamster Model	SARS-CoV-2	Abdelnabi et al. (2021) and Rosenke et al. (2021)

MERS-CoV: Middle East respiratory syndrome-related coronavirus; SARS-CoV-2: The virus, severe acute respiratory syndrome coronavirus 2; SARS-CoV: severe acute respiratory syndrome-related coronavirus: LoM: Lung only Mice

As a ribonucleoside analog, NHC has a reputation for affecting mitochondrial replication and function. However, in vitro, NHC did not cause significant mitochondrial toxicity or impair mitochondrial function (Sticher et al. 2020). After seven days of treatment with EIDD-2801, ferret lung tissue did not show any significant changes in nuclear or mitochondrial message transition rates (Toots et al. 2019).

Evidence suggests that the NHC exerts antiviral effects by selective introduction of mutations in only viral RNA keeping host RNA untouched, indicating a high genetic barrier to NHC resistance (Agostini et al. 2019). Furthermore, its antiviral ability is supported by its similarity to other potent antivirals, such as cytidine (Mestres 2020).

### The investigation by clinical trials on the patient of COVID-19

After the successful completion of phase I safety trials (NCT04392219) for EIDD-2801 (Molnupiravir), a drug that inhibits RNA virus replication, advanced multi-center clinical trials were propelled to evaluate the efficacy of the drug. Merck & Co, on the other hand, has yet to reveal the dosages consumed and clinical pharmacokinetic data (2021). The company ran phase II and III clinical trials, involving over 600 COVID-19 patients, using a randomized strategy to administer molnupiravir 200 mg, 400 mg, 800 mg, or placebo twice daily for five days. The trials was to show if the treatment leads to long-term recovery or reduces the number of hospital admissions or deaths (2021; Jayk Bernal et al. 2021; Mahase 2021). The companies also reported findings on one secondary objective from the Phase 2a study, showing a reduction in time (days) to the negativity of infectious virus isolation in nasopharyngeal swabs from participants with symptomatic SARS-CoV-2 infection, as determined by isolation in Vero cell line culture (News 2021). In a Phase III trial, it was reported that early treatment with molnupiravir lessened the risk of hospitalization or death in at-risk, unvaccinated adults with COVID-19 (Singh et al. 2021) (Jayk Bernal et al. 2021). All the clinical trials are summarized in Table 3. The most often reported adverse effects were nausea, diarrhoea, and headache—all of which were minor in intensity. Nobody withdrew from the trial.

**Table 3:** A list of clinical trials of molnupiravir registered on www.ClinicalTrial.gov

Identification no.	Title	Expected participants/dosage	Region
NCT04392219	COVID-19 First in human study to evaluate safety, tolerability, and pharmacokinetics of EIDD-2801 in healthy volunteers	130/Single dose or two single dosages of Molnupiravir orally	United Kingdom
NCT04405570	A safety, tolerability and efficacy of molnupiravir (EIDD-2801) to eliminate infections virus detection in persons with COVID-19	204/Molnupiravir twice daily for 5 days	United States, Multicounty
NCT04405739	The safety of molnupiravir (EIDD-2801) and its effect on viral shedding of SARS-CoV-2 (END-COVID)	96/EIDD-2801 orally twice daily for 5 days	United States, Multicounty
NCT04746183	AGILE (Early Phase Platform Trial for COVID-19)	600/Molnupiravir orally 10 dosage	United Kingdom
NCT04575584	Efficacy and safety of molnupiravir (MK-4482) in hospitalized adult participants with COVID-19 (MK-4482-001)	304 200 mg or 400 mg or 800 mg Molnupiravir orally every 12 h for 5 days	Brazil, Canada, Chile, Colombia, France, Israel, Italy, Korea, Poland, Russia, South Africa, Spain, Ukraine, United States, United Kingdom
NCT04575597	Efficacy and safety of molnupiravir (MK-4482) in non-hospitalized adult participants with COVID-19 (MK-4482-002)	1450/Molnupiravir administered as capsule orally every 12 h for 5 days	United States, Multicounty
NCT04939428	MK-4482 for Prevention of Coronavirus Disease 2019 (COVID-19) in Adults (MK-4482-013) (MOVe-AHEAD)	1332/4 Molnupiravir 200 mg oral capsule	Multicounty

## Conclusions

Molnupiravir is a bioactive oral form of a highly potent ribonucleoside analog that halts the replication of multiple RNA viruses, including SARS-CoV-2, responsible for COVID-19. The drug possesses strong activity against multiple coronaviruses for treatment and prophylactically, as revealed in animal studies as well as in vitro studies using cell culture. EIDD-2801 has been shown to recover pulmonary function and mitigate the amount of virus in the lung. Also, EIDD-2801 has been shown to be effective against pneumovirus, influenza, chikungunya virus, orthomyxovirus, alpha virus, and Ebola virus, so it can help fight off other viruses that can cause sickness. Molnupiravir offers a fascinating feature to have a high barrier to resistance as compared with another ribonucleoside analog of the same class (Remdesivir) that forces viruses to rapidly generate mutants, remain unaffected by the same drug. This limits the widespread use of remdesivir-like drugs and necessitates intravenous administration. But, Molnupiravir, provides the option that the drug can be taken at home rather than being restricted to hospital use only. Clinical applications of molnupiravir to quickly treat COVID-19 patients and prevent SARS-CoV-2 transmission may be expected to benefit both individual and public health.

## Data Availability

Not applicable.

## Abbreviations

µM: Micromole
BVDV: Bovine viral diarrhea virus
Calu-3: Human bronchial epithelial cells
Coronavirus MHV-A59: Coronavirus murine hepatitis virus A-59
EC 50: Half maximal effective concentration
EIDD-1931: β-DN^4^-hydoxycytidine
EIDD-2801: Molnupiravir
HAE: Human airway epithelial cells
IC 50: Half maximal inhibitory concentration
LoM: Lung only Mice
MERS-CoV: Middle East respiratory syndrome-related coronavirus
NHC: β-DN^4^-hydoxycytidine
SARS-CoV: Severe acute respiratory syndrome-related coronavirus
SARS-CoV-2: The virus, severe acute respiratory syndrome coronavirus 2
Vero E6: African green monkey kidney epithelial cells
VEEV: Venezuelan equine encephalitis virus
WHO: World Health Organization
